# Evaluation of cavitation enhancements in low-boiling point (< −2°C) perfluorocarbon nanodroplet and microbubble mixtures using therapeutic ultrasound pulses

**DOI:** 10.1016/j.ultsonch.2026.107850

**Published:** 2026-04-13

**Authors:** Haseeb Khan, Paul A. Dayton, Zhen Xu, Xiaoning Jiang, Jinwook Kim

**Affiliations:** a School of Mechanical Engineering, Kyungpook National University, Daegu 41566, the Republic of Korea; b The Lampe Joint Department of Biomedical Engineering, The University of North Carolina at Chapel Hill, Chapel Hill, NC 27599, USA; c Department of Biomedical Engineering, University of Michigan, Ann Arbor, MI 48109, USA; d Department of Mechanical and Aerospace Engineering, NC State University, Raleigh, NC 27695, USA

**Keywords:** Cavitation, Pulsed ultrasound, Passive cavitation detection, Microbubble, Nanodroplet

## Abstract

This study investigates the phenomenon of cavitation enhancement by using a combination of low-boiling-point (< −2°C) perfluorocarbon nanodroplets (NDs) and microbubbles (MBs) following the application of therapeutic ultrasound pulses. While MBs and NDs individually exhibit distinct cavitation behaviors, their combined effects are not well understood. We hypothesize that integrating MBs with NDs enhances cavitation via synergistic interactions between gas-filled and liquid cores, increasing both cavitation dose and duration. In-vitro experiments with passive cavitation detection were performed under various conditions using six agents: decafluorobutane MB (DFB MB), DFB ND, octafluoropentane ND (OFP ND), and their combinations. Ultrasound pulses (1 MHz, 1.2–7.5 MPa peak negative pressure) were used to quantify stable and inertial cavitation. At lower pressures (1.2–4.6 MPa), individual and combination treatments produced comparable stable cavitation. However, at higher pressures (6.3–7.5 MPa), DFB MB + DFB ND yielded more stable cavitation effects than those induced by DFB MB alone (by 48.8–100.7%), DFB ND alone (by 34.4–38.1%), and OFP ND alone (by 97.6–104.1%). For inertial cavitation, combinations enhanced activity by 13.4–100.7% (vs. DFB MB), 32.3–38.1% (vs. DFB ND), and up to 104.1% (vs. OFP ND) across all excitation pressure cases. Temporally, individual agents showed two distinct peaks that intensified with pressure, whereas combination agents exhibited a smoother, merged response from overlapping cavitation. These results suggest that MB–ND combinations can enhance cavitation relative to MBs or NDs alone, with the magnitude of enhancement depending on the specific formulation and excitation pressure. We expect our findings motivate further development of MB–ND–based strategies for spatiotemporally controlled and efficient therapeutic ultrasound applications, including sonothrombolysis, tumor ablation, and targeted drug delivery.

## 1 Introduction

Acoustic cavitation involves nucleation, growth, oscillation, and collapse of microbubbles in a liquid driven by acoustic pressure waves [1], [2]. This phenomenon appears in two primary forms: stable cavitation, where bubbles oscillate nondestructively over multiple acoustic cycles and can generate fluid motion and mechanical agitation through microstreaming, and inertial cavitation, where bubbles undergo rapid growth followed by collapse, producing violent mechanical effects such as microjets and shock waves [2], [3]. The dynamic nature of acoustic cavitation has been actively utilized in various applications, including sonochemistry [4], ultrasonic therapies [5], ultrasonic cleaning [6], and biological sample processing [7].

Cavitation-induced mechanical effects are influenced by multiple factors, including peak negative pressure (PNP), acoustic radiation force, and subsequent streaming-induced agitation. Typically, acoustic cavitation requires a very high-PNP (> 10 MPa), which is commonly achieved using short bursts rather than continuously, enabling the generation of strong negative pressure during each cycle while minimizing thermal accumulation in the surrounding medium. According to classical nucleation theory, once the rarefaction pressure surpasses the liquid’s tensile strength, the formation of critical-sized vapor nuclei becomes thermodynamically favorable, thereby initiating the cavitation process [8], [9]. In biomedical applications, achieving cavitation at a confined zone by lower PNP levels is preferable since precision and safety are crucial. The challenge is the achievement of precise spatiotemporal control over cavitation events while maintaining therapeutic efficacy with minimized tissue damage [10], [11].

One strategy to lower the cavitation threshold and improve spatiotemporal control involves the use of cavitation nuclei, such as microbubbles (MBs), nanodroplets (NDs), or synthesized nanoparticles [12]. MBs are typically composed of perfluorocarbon gas-filled lipid, polymer, or protein shells, and these particles have been employed as 1) contrast agents in ultrasound imaging and as 2) cavitation enhancers in therapeutic ultrasound. Their ability to reduce the threshold pressure amplitude for cavitation initiation and enhance cavitation dose has made them versatile in applications of sonothrombolysis, targeted drug delivery, and blood–brain barrier opening [13], [14], [15]. However, MBs have a relatively short lifespan in circulation owing to their rapid dissolution, which limits their practical uses in sustained cavitation-based applications [16], [17].

Phase-change NDs have emerged as promising cavitation agents due to their unique properties. These sub-micron-sized liquid droplets stabilized by a lipid or polymeric shell involve phase transition into gas MBs through heat or acoustic pressure. Thus, ultrasonic pressure wave-induced vaporization has been actively utilized as a useful mechanism in therapeutic ultrasound applications, as NDs have several advantages when compared with MBs including longer circulation times, higher stability, and enhanced extravasation into tissue microenvironments [18], [19]. Their small sizes possibly enable them to penetrate deeper into biological tissues, which makes them attractive candidates for precision-focused cavitation applications.

Phase-change NDs typically require a relatively high threshold PNP to trigger cavitation compared to MBs. Conventional perfluorocarbon-core nanodroplets, such as perfluoropentane (PFP, C_5_F_12_) and perfluorohexane (PFH, C_6_F_14_) NDs have boiling points ranging from 27°C to 56°C. Consequently, they require high-PNP excitation (>∼7 MPa) to vaporize under short-pulsed (<5 cycles) conditions [20], [21]. In contrast, low-boiling-point NDs with a decafluorobutane (DFB, C_4_F_10_, −2°C) or octafluoropropane (OFP, C_3_F_8_, −37°C) gas core exhibit considerably lower vaporization thresholds (<3 MPa for 1 MHz pulses) enabling their activation at modest PNP levels [22], [23]. This characteristic makes DFB- or OFP-core NDs particularly suitable for biomedical applications where lower acoustic intensities are desired to minimize collateral damage, thereby facilitating precise and efficient cavitation-enhanced ultrasound therapies [24].

Previous studies have highlighted the distinctive characteristics of low-boiling-point NDs compared with MBs made of the same shell-gas core composition in ultrasound therapies. For example, under modest-mechanical index (MI > 1) and pulsed excitation conditions, nanodroplet (ND)-mediated cavitation produces a higher and longer-lasting cavitation level than that produced by microbubbles (MB)-mediated cavitation [10], [25], while under low-MI (<0.3) conditions, the cavitation dose is greater when MBs are used instead of NDs [26]. Additionally, the vaporization threshold pressure for droplets is lower at higher frequency excitations owing to superharmonic focusing effects [27], whereas bubble rupture tends to occur more easily at lower frequencies [28]. Although each cavitation agent exhibits distinct advantages, only a few studies have explored their combined effects when MBs and NDs are used simultaneously [29], [30], [31]. While some studies have utilized ND-coated MB particles or multistep excitation protocols to capitalize on the sequential dynamics of phase change and cavitation, particularly in applications such as sonothrombolysis or targeted drug delivery [31], [32], [33], the integrative potential of combining these two agents has not yet been systematically evaluated.

We hypothesize that integrating MBs with low-boiling-point NDs will provide synergistic advantages, surpassing the efficacy of either agent alone. The presence of suspended gas cores from microbubbles in proximity to NDs may enhance cavitation dynamics by altering the inertial cavitation environment, leading to a more robust and sustained cavitation response. We anticipate that the synergistic interaction increases the cavitation dose at the same excitation energy; therefore, the MBs–NDs mixture may possess potential in improving the efficiency and effectiveness of ultrasound therapy. To test this hypothesis, we designed an in-vitro study utilizing mixtures of DFB MBs and low-boiling-point NDs, including both the DFB and OFP formulations. By systematically evaluating their cavitation behavior under passive cavitation detection and controlled ultrasound exposure conditions at an in vitro flow system, we aim to elucidate the potential benefits of combining these agents. Our investigation focuses on key parameters such as cavitation level and persistence, which are critical for optimizing therapeutic applications. Unlike prior studies that have primarily examined each agent independently, this study highlights the cooperative interplay between gas- and liquid-based cavitation nuclei under therapeutic ultrasound. By establishing a mechanistic link between agent combinations and improved cavitation performance, this study aims to provide useful guideline to the design of acoustically responsive contrast agents.

## 2 Materials and methods

### 2.1 Experimental setup

**Fig. 1:**
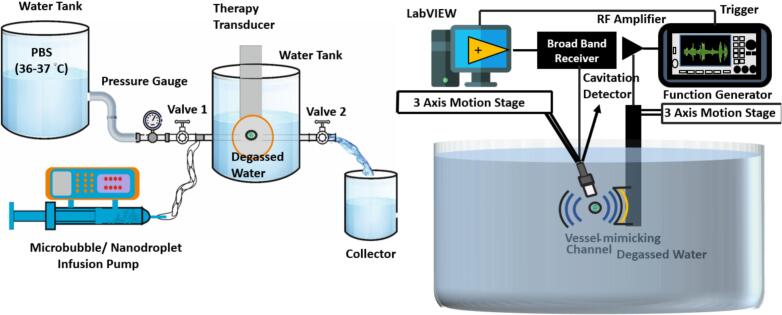
Experimental setup. (a) Front view showing the vessel-mimicking channel filled with phosphate-buffered saline (PBS, 36–37°C) and MB/ND suspension, (b) Side view including the radio frequency (RF) amplifier and 3-axis motion stage.

For this study, we utilized the previously developed in-vitro flow model integrated into a therapeutic ultrasound experimental setup (Fig. 1) [26]. This femoral vein phantom (Tygon S3 E-3603, Saint-Gobain Performance Plastics, Akron, OH, USA) has a wall thickness of 0.8 mm and an inner diameter of 6.4 mm. Flow conditions were kept consistent by fixing the reservoir height and valve opening, which maintained a steady flow rate of 80 ± 15 mL/min (Fig. 1), while the water temperature was controlled as 36 ± 0.5°C.

We used a 1-MHz concave transducer as the excitation source (H-131, Sonic Concepts, Inc., Bothell, WA, USA) with an aperture of 33 mm and a focal length of 35 mm. Raster scanning with a hydrophone (HNA-400, Onda Co., Sunnyvale, CA, USA) characterized the transducer and yielded a full-width-at-half-maximum of 2.4 mm in the lateral direction and 17.4 mm in the axial direction. The focused transducer was driven by an arbitrary function generator (AFG3101, Tektronix Inc., Beaverton, OR, USA) in conjunction with a 55-dB radio frequency amplifier (A150, E&I, Rochester, NY, USA) [10], [26]. The pulsing conditions were the following: 1-MHz, 10-cycle pulses were used at a pulse repetition frequency (PRF) of 2 Hz, resulting in a duty cycle of 0.002%. The PNP was set at five levels: 1.2, 3.1, 4.6, 6.3, and 7.5 MPa. A previously reported computerized 3‑axis motion-stage setup (Newport XPS, Irvine, CA, USA) controlled by LabVIEW (National Instruments, Austin, TX, USA) was used to reproducibly align the acoustic focus of the 1‑MHz concave transducer with the center of the vessel‑mimicking flow channel, thereby ensuring consistent ultrasound exposure geometry across all experimental conditions [26].

Cavitation signals were acquired using a 5-MHz piston transducer (V309, Panametrics, Waltham, MA, USA) positioned 50 mm away from the tube (which also served as the ultrasound focus) at an angle of 45° (Fig. 1B). Signals were captured with a broadband receiver (BR-640A, Ritec, Inc., Warwick, RI, USA) and recorded via LabVIEW. For each experimental condition, 30 sets of time-domain signals were saved, with each set digitized using 16,000 data points over a time window spanning 20 μs to 100 μs when the excitation pulse was triggered at 0 s.

### 2.2 Fabrication of nanodroplets and microbubbles

We prepared three distinct in-house cavitation agents following the previously reported protocols: 1) DFB MBs, 2) DFB NDs, and 3) OFP NDs [10], [15]. The lipid solution used to prepare the agents was based on widely used protocols for low-boiling-point perfluorocarbon MBs and phase-change NDs. The shell lipids consisted of 1,2-distearoyl-*sn*-glycero-3-phosphocholine (DSPC) and 1,2-distearoyl-*sn*-glycero-3-phosphoethanolamine-N-methoxy(polyethylene glycol)-2000 (DSPE-PEG2000) at a 9:1 M ratio, formulated at a total lipid concentration of 1.0 mg/mL in an excipient composed of phosphate-buffered saline (PBS), propylene glycol, and glycerol (16:3:1, equivalent to 80% PBS, 15% propylene glycol, 5% glycerol) [25], [34], [35]. MB and ND stocks were prepared separately and combined immediately prior to experiments to achieve the intended number concentration of each component in the mixture. Briefly, a 3 mL vial was loaded with 1.5 mL of lipid solution. The headspace air was then replaced with DFB gas (FluoroMed LP, Round Rock, TX, USA), and the vial was shaken using a Vialmix (Lantheus Medical Imaging, North Billerica, MA, USA) to produce lipid-shelled MBs with a DFB core. These MBs were subsequently condensed at −13°C while pressurizing the vial headspace until condensation was observed, resulting in the formation of liquid-DFB core NDs. The preparation procedure for OFP NDs was identical to that of DFB NDs, except that OFP gas (FluoroMed LP, Round Rock, TX, USA) was used instead of DFB gas. The resulting MB particles exhibited a mean diameter of 1.1 μm (0.6–4 μm) and a concentration of 1 × 10^10^/mL, as measured by an Accusizer 780 (Particle Sizing Systems, Santa Barbara, CA, USA). The ND particle sizes and concentrations were determined using an Accusizer FX-Nano (Entegris, Billerica, MA, USA), yielding average DFB ND sizes of 0.33 μm (0.25–0.6 μm) with a concentration of 1.0 × 10^10^/mL, and OFP ND sizes of 0.32 μm with a concentration of 9 × 10^9^/mL. For passive cavitation testing, three vials of each cavitation agent were prepared for three repetitions of the data acquisition.

### 2.3 Passive cavitation detection test procedure

We conducted passive cavitation detection (PCD) using a flow model to compare the cavitation characteristics of a cavitation agent mixture versus individual agent infusions. Six infusion conditions were tested: (1) DFB MB, (2) DFB ND, (3) OFP ND, (4) DFB MB + DFB ND, (5) DFB MB + OFP ND, and (6) DFB ND + OFP ND, comprising three single-agent and three mixture-agent cases.

For single-agent infusions, we prepared a 1 mL syringe containing 0.9 mL of phosphate-buffered saline (PBS, Fisher Scientific, Pittsburgh, PA, USA) and added 0.1 mL of the selected cavitation agent (DFB MB, DFB ND, or OFP ND). The syringe was gently rolled by hand to achieve an approximate particle concentration of 1 × 10^9^ particles/mL. For mixture-agent cases, we added 50 μL of each selected cavitation agent to the PBS-filled syringe, maintaining a consistent 1:10 vol ratio of cavitation agent to PBS across all test conditions. Given that the estimated particle concentration in each vial was 10 ± 1 billion particles/mL, we maintained that both single-agent and mixture-agent syringes contained a similar effective cavitation agent concentration. The prepared syringe was then connected to an infusion tube and permanently catheterized into the flow model (Fig. 1A). During PCD testing, each cavitation agent was continuously infused at a rate of 80 μL/min using a micropump (PHD2000, Harvard Apparatus, Holliston, MA, USA). To minimize cavitation effects from residual agent particles from previous tests, a PBS-filled syringe was prepumped into the channel at a rate of 0.2 mL/min for 1 min before each test case.

In this study, PCD signals were post processed and analyzed using a previously established method to calculate the stable cavitation level (SCL) and inertial cavitation level (ICL). Stable cavitation refers to the gentle oscillation of bubbles in response to ultrasound without causing collapse, emitting harmonic signals detectable at harmonic and subharmonic frequencies. In contrast, inertial cavitation involves the rapid growth and violent collapse of bubbles, releasing high-energy shock waves and generating broadband noise across a broad frequency range [10], [36]. To quantify the SCL, the second harmonic component (2*f*_0_) was extracted using a 5th-order Butterworth bandpass filter within the range of 1.5–2.5 MHz, where *f*_0_ represents the therapy wave’s operating frequency (1 MHz) [37]. We also acquired agent-free (ultrasound-only) baseline data under the same acoustic and geometric conditions, however, these baseline measurements were not subtracted from the SCL calculations. The baseline values reflect the combined effects of propagation- and system-related nonlinearities including second-harmonic components directly received from the excitation pulses. Accordingly, we interpret the 2*f*_0_-band metric relative to this agent-free baseline and emphasize comparisons across agents under identical excitation conditions, rather than treating the absolute 2*f*_0_ magnitude as a definitive, stand-alone indicator of stable cavitation [10], [26].

The ICL was determined by analyzing the broadband noise within the 3–7 MHz range. As superharmonic components (3*f*_0_, 4*f*_0_, 5*f*_0_, 6*f*_0_, and 7*f*_0_) are also associated with nonlinear bubble oscillations linked to stable cavitation, signals within ±0.2 MHz of each superharmonic were excluded from the ICL calculation. Cavitation levels were quantified by calculating the area under the curve (AUC) of the filtered frequency spectrum. It is important to note that SCL includes signals from the directly received excitation that are unrelated to bubble dynamics. Owing to the narrowband excitation and the nonlinear effects of strong intensity waves (with peak negative pressures up to 7.5 MPa), the cavitation detector recorded higher harmonics. To ensure accurate ICL measurements, content around the harmonic peaks (3*f*_0_, 4*f*_0_, 5*f*_0_, 6*f*_0_, and 7*f*_0_, each within a ±0.2 MHz range) was selectively removed from the AUC calculation [10], [26].

### 2.4 Statistical analysis

For the cavitation efficacy test, six different treatment cases were evaluated. For each case, 30 data lines were analyzed (n = 30) to quantify passive cavitation levels. Statistical significance across treatments was determined using one-way ANOVA [35] followed by Tukey’s Honest Significant Difference (HSD) test [34], [40] (p < 0.05). The p-values for all treatment comparisons are provided in the Supplementary Material (Section 2).

## 3 Results

**Fig. 2:**
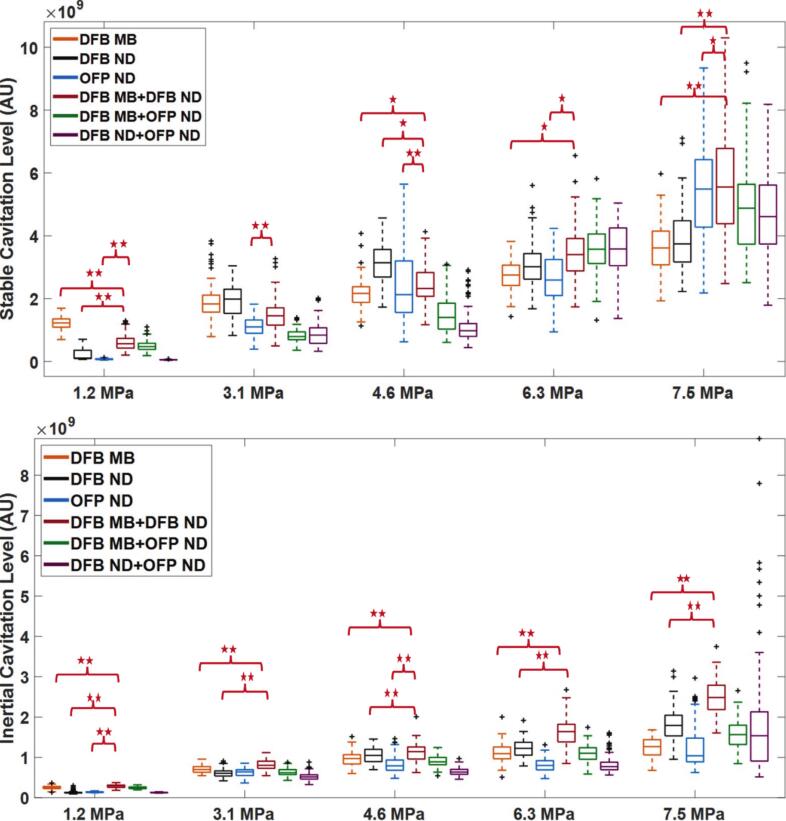
Stable (A) and inertial (B) cavitation across five peak negative pressures (1.2–7.5 MPa) under six conditions (DFB MB, DFB ND, OFP ND, DFB MB + DFB ND, DFB MB + OFP ND, DFB ND + OFP ND). For simplicity, only the comparisons of individual treatments with the one mixture were highlighted in the graphs. A single asterisk (*) indicates significance (p < 0.05), and a double asterisk (**) indicates p < 0.01.

In this study, we hypothesized that combining MBs and NDs could influence cavitation activity through interactions between their gas and liquid phases. The in-vitro PCD measurements revealed pressure-dependent variations in cavitation activity among the tested agents and their combinations (Fig. 2A and B). Some mixtures exhibited higher stable cavitation levels at elevated pressures (6.3–7.5 MPa), while inertial cavitation activity was observed over a broader pressure range (3.1–7.5 MPa). These observations describe the overall cavitation behavior across the tested pressure conditions rather than indicating uniform enhancement for all combinations.

**Fig. 3:**
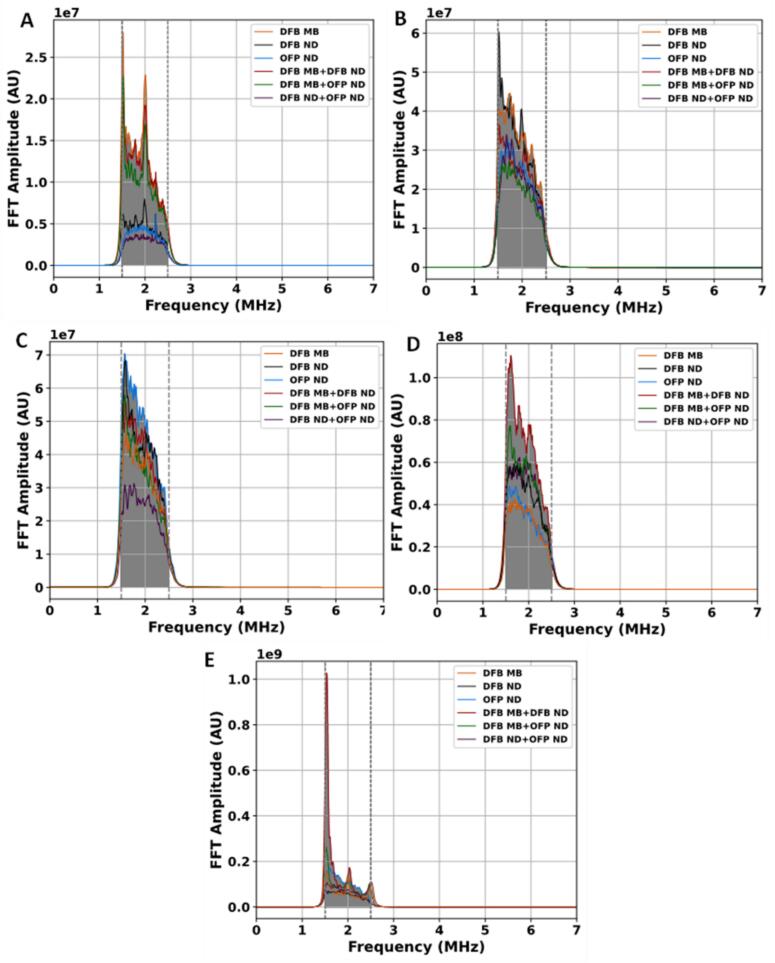
Preprocessed FFT spectra used for stable cavitation analysis. Untreated spectra are shown in the Supplementary Material (Fig. S1).

**Fig. 4:**
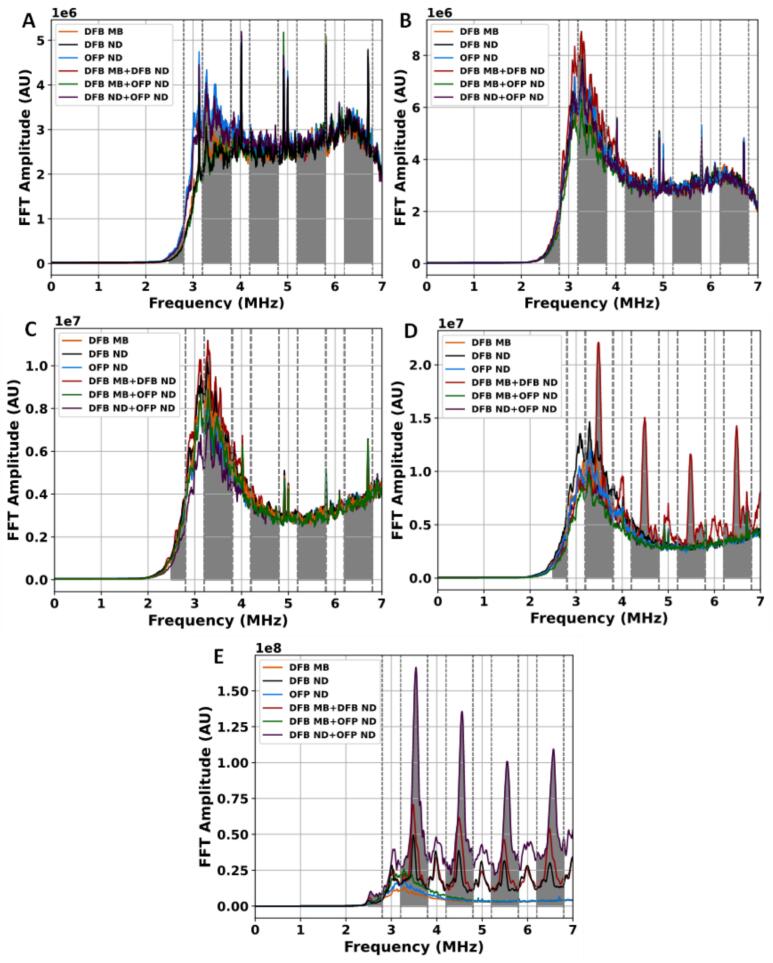
Preprocessed FFT spectra used for inertial cavitation analysis. The untreated spectra are shown in the Supplementary Material (Fig. S1).

Additionally, when comparing all six cavitation agent cases under the same pressure and experimental conditions, differences in stable and inertial cavitation levels were observed among the individual agents and their combinations. These differences were also reflected in the corresponding fast Fourier transform (FFT) spectral amplitudes shown in Fig. 3, Fig. 4. However, the relatively large variability observed in several cases resulted in overlapping distributions between treatments, indicating that the results should be interpreted as pressure-dependent tendencies rather than strictly distinct cavitation responses. The overall comparison of SCL and inertial cavitation level ICL across the six cavitation agent cases at different PNP inputs is summarized in Fig. 2A and B.

At a pulsed excitation of 1.2 MPa, the DFB MB treatment exhibited the highest SCL (Fig. 2A), highest FFT amplitude (Fig. 3A), and the largest AUC (Table 1, row 1). This treatment yielded a broad distribution, indicating substantial cavitation activity compared with other cases. The DFB MB + DFB ND combination followed, demonstrating moderate stable cavitation and FFT amplitude, higher than DFB ND, and OFP ND alone but lower than DFB MB alone (Figs. 2A and 3).

**Table 1:** Averaged areas under curve (AUCs) for stable cavitation level (SCL) calculation. All values, except for PNP column, are ×10^7^ (arbitrary unit).

PNP (MPa)	DFB MB	DFB ND	OFP ND	DFB ND + DFB MB	DFB ND + OFP ND	DFB MB + OFP ND
1.2	1.28	0.46	0.38	1.17	0.31	1.02
3.1	3.14	3.15	2.33	2.45	2.21	1.96
4.6	3.63	4.42	5.06	4.18	2.52	3.74
6.3	3.48	4.76	3.76	7.25	5.16	5.41
7.5	5.74	7.31	11.30	18.90	7.65	10.90

Among individual treatments, DFB ND and OFP ND exhibited lower cavitation levels, with OFP ND showing minimal variability and a low median. The DFB ND + OFP ND combination yielded the lowest SCL, FFT amplitude, and AUC value, with a narrow distribution and minimal variability. The ICL results at 1.2 MPa exhibited a similar trend to the SCL results; the DFB MB, DFB MB + DFB ND, and DFB MB + OFP ND cases exhibited higher ICL values than those of the ND-alone cases, indicating MB cavitation at a pressure field with an MI of 1.2, which is sufficient for DFB MB rupture as reported previously (MI > 0.3) [11], [28]. Additionally, lower cavitation levels were observed in DFB ND and OFP ND, with DFB ND slightly surpassing OFP ND, both exhibiting limited variability. The DFB ND + OFP ND combination demonstrated the lowest inertial cavitation level and FFT amplitude (Fig. 4), indicating limited vaporization and rupture during the 10-cycle burst excitation. Statistically, most treatment groups showed significant differences, except for DFB MB vs. DFB ND (p = 0.260), DFB MB + DFB ND vs. DFB ND + OFP ND (p = 0.827) in stable cavitation as shown in the Table 1S (Supplementary Material), and DFB ND vs DFB ND + OFP ND (p = 0.088) in inertial cavitation refer to Table 1s in the Supplementary Material.

At 3.1 MPa, the DFB ND treatment exhibited the highest SCL, as shown in Fig. 2, with the largest FFT amplitude observed in Fig. 3B. The DFB MB case followed with a slightly lower SCL, characterized by a broad range of outliers. The combination of DFB MB and DFB ND resulted in a lower SCL than either individual treatment. The remaining treatments, including OFP ND, DFB MB + OFP ND, and DFB ND + OFP ND, demonstrated comparatively lower SCL values, with the lowest median and minimal variability, as reflected in the AUC values in Table 1 (row 2). For inertial cavitation at the same pressure, the DFB MB + DFB ND combination produced the highest levels, with a broad distribution indicative of enhanced cavitation activity. This combination also exhibited the highest peak FFT amplitude. The DFB MB treatment followed, showing a similar distribution but slightly lower ICL levels. Moderate inertial cavitation was observed in DFB ND and OFP ND, with DFB ND displaying a slightly higher median and greater variability than OFP ND. Among the combination treatments, DFB MB + OFP ND exhibited moderate cavitation levels, while DFB ND + OFP ND yielded the lowest levels, with minimal variability and a few outliers. At this PNP, the majority of treatment groups exhibited statistically significant differences in both stable and inertial cavitation as shown in Table S2 for stable and Table S7 for inertial cavitation in the supplementary material. However, a subset of comparisons lacked statistical significance. In the stable cavitation analysis, DFB MB was statistically indistinguishable from both DFB ND (*p* = 0.519) and DFB MB + DFB ND (*p* = 0.053). Likewise, DFB MB + OFP ND showed no statistically meaningful difference relative to DFB ND (*p* = 0.148) and DFB MB + DFB ND (*p* = 0.859). The comparison between DFB ND and DFB MB + DFB ND also failed to yield statistical separation (*p* = 0.637). Similarly, in the inertial cavitation analysis, similar trends were evident. DFB MB + OFP ND showed no statistically meaningful difference when compared to either DFB ND (*p* = 0.671) or DFB MB + DFB ND (*p* = 0.583). Additionally, the responses from DFB MB + DFB ND and OFP ND (*p* = 0.104), as well as from DFB ND + OFP ND and OFP ND (*p* = 0.075), were statistically comparable.

At 4.6 MPa, the OFP ND treatment exhibited the highest SCL (Fig. 2) and the largest FFT amplitude (Fig. 3C), with a broad distribution and distinctive variability, indicating notable cavitation activity across samples. The DFB ND treatment followed, showing the second highest SCL, while the DFB MB + DFB ND combination ranked third. The DFB MB and DFB MB + OFP ND treatments demonstrated moderate SCL levels, with fewer outliers and narrower distributions compared with those of DFB ND and OFP ND. The lowest SCL was observed in the DFB ND + OFP ND case, characterized by minimal variability. For the ICL at the same pressure, the DFB MB + DFB ND combination exhibited the highest level, with the highest FFT amplitude and the largest AUC values, as shown in Figs. 2 and 4C, and as listed in Table 2 (row 3). The DFB ND treatment followed, displaying high levels with slightly lower variability. The DFB MB and OFP ND treatments exhibited moderate ICL levels, with DFB MB exhibiting a slightly broader distribution. Meanwhile, the DFB MB + OFP ND and DFB ND + OFP ND combinations recorded the lowest inertial cavitation levels.

**Table 2:** Averaged AUCs for inertial cavitation level (ICL) calculations. Except for PNP column, all the presented values are ×10^7^ (arbitrary units).

PNP (MPa)	DFB MB	DFB ND	OFP ND	DFB ND + DFB MB	DFB ND + OFP ND	DFB MB + OFP ND
1.2	0.99	0.96	0.98	1.02	0.97	0.99
3.1	1.35	1.17	1.32	1.53	1.20	1.34
4.6	3.33	3.45	3.32	3.52	3.14	3.24
6.3	2.95	2.69	2.61	3.64	2.56	2.39
7.5	2.71	10.40	3.17	13.00	26.70	3.94
						

Regarding statistical significance at 4.6 PNP, as detailed in Supplementary Tables S3 and S8, respectively. In the stable cavitation analyses, a few group comparisons yielded non-significant differences. Specifically, DFB MB + OFP ND was statistically comparable to DFB ND (*p* = 0.957) and to DFB MB + DFB ND (*p* = 0.0895). Additionally, DFB ND showed no statistically meaningful difference from DFB MB + DFB ND (*p* = 0.0224), though this value borders the significance threshold and may warrant cautious interpretation. In contrast, the inertial cavitation analysis revealed more distinct separations across treatments. However, the pairwise comparison between DFB MB + OFP ND and DFB MB + DFB ND failed to reach significance (*p* = 0.994), indicating comparable inertial responses. All other comparisons in both stable and inertial cavitations showed significant differences (*p* < 0.05), with most falling below *p* < 0.001, confirming robust treatment-induced effects at this pressure level.

At 6.3 MPa and 7.5 MPa, the DFB MB + DFB ND combination consistently exhibited the highest SCLs, as shown in Figs. 2A, 3D, and E. This was followed by DFB MB + OFP ND and DFB ND + OFP ND, both exhibiting slightly lower SCL levels. DFB ND showed moderate SCL, FFT amplitude, and AUC values, with reduced variability, indicating a consistent but lower response. OFP ND demonstrated lower SCLs than DFB ND, with a narrower distribution and fewer outliers. DFB MB recorded the lowest stable levels, with minimal variability, suggesting limited cavitation activity. For inertial cavitation at these pressures, DFB ND + OFP ND exhibited the highest levels (Fig. 2B), with the strongest FFT amplitude and a broad range with multiple outliers. DFB MB + DFB ND followed, displaying substantial inertial cavitation with a narrower distribution. DFB MB recorded the lowest ICLs, characterized by minimal variability, indicating relatively limited cavitation activity.

Across the higher-pressure levels of 6.3 MPa and 7.5 MPa, most treatment groups demonstrated significant differences in both stable and inertial cavitation responses, as detailed in Supplementary Tables S4–S5 and S9–S10. Nevertheless, certain group comparisons yielded statistically comparable results. In the stable cavitation analysis at 6.3 MPa, the response from DFB MB + OFP ND was similar to that of DFB ND (p = 0.0508), and DFB MB + DFB ND showed no statistical separation from DFB ND (p = 0.951) or from DFB ND + OFP ND (p = 0.477). At 7.5 MPa, DFB MB + OFP ND again produced responses statistically aligned with both DFB ND (p = 0.4032) and DFB MB + DFB ND (p = 0.6414), while DFB MB + DFB ND and DFB ND + OFP ND remained comparable (p = 0.800). A similar trend was evident in inertial cavitation. At 6.3 MPa, DFB MB + OFP ND was statistically aligned with DFB MB + DFB ND (p = 0.6414), and DFB ND produced responses similar to DFB MB + DFB ND (p = 0.3422). At 7.5 MPa, these comparable patterns persisted, with DFB MB + OFP ND showing similarity to DFB MB + DFB ND (p = 0.3422), and DFB MB + DFB ND statistically matching DFB ND + OFP ND (p = 0.800). These results suggest convergence in cavitation behavior among droplet-based or combination treatments at elevated pressure levels.

**Fig. 5:**
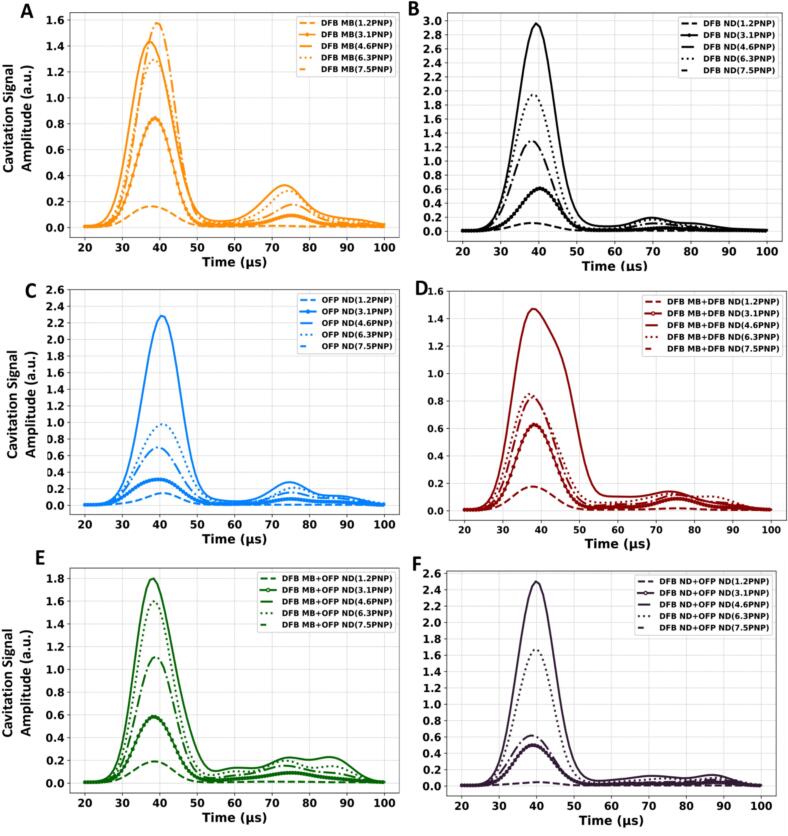
Cavitation signals in the time domain. a) DFB MB, 2) OFP ND, 3) DFB ND, 4) DFB MB + DFB ND, 5) DFB MB + OFP ND, and 6) DFB ND + OFP ND.

We also investigated to identify specific patterns and examine the effects of pressure on each treatment in the time domain, as shown in Fig. 5. By analyzing cavitation signals over time, we observed that each treatment exhibits a characteristic initial response duration. At lower pressures, the cavitation signals comprised a sharp peak initially, followed by a gradual decline. As pressure increases, this initial peak becomes more pronounced, indicating an intensified cavitation response. DFB MB and OFP ND exhibit a distinct initial peak that strengthens with increasing pressure, followed by a secondary wave packet after a short delay (15 μs on average). DFB ND follows a similar pattern but with smaller amplitudes in both primary and secondary wave packets compared with the DFB MB and OFP ND cases. Combination treatments (DFB MB + DFB ND and DFB MB + OFP ND) show a broader initial peak with an extended duration, indicating more sustained and subsequent cavitation responses. The broader response remains consistent as pressure increases, suggesting a modified temporal pattern for combination treatments. While the cavitation signal amplitude increased with increasing pressure across most treatments, DFB MB behaved differently, displaying high amplitudes even at lower pressures (≤4.6 MPa) unlike other treatments that required higher pressures to reach similar levels (see Supplementary Materials S3). This behavior likely results owing to the lower rupture threshold (MI < 0.3) of DFB MB compared with that of the ND (MI > 1) [26], [28].

## 4 Discussion

This study investigated how combinations of microbubbles (MBs) and nanodroplets (NDs) influence cavitation behavior under pulsed ultrasound excitation. The pressure-dependent behavior of MB–ND mixtures can be interpreted in terms of competing mechanisms that alter 1) the effective acoustic field experienced by nuclei and 2) the available bubble population within the focal region. Oscillating microbubbles and vaporized droplets can strongly scatter and absorb acoustic energy; when a sufficiently dense bubble population forms, the incident field can be partially attenuated, thereby reducing the effective negative pressure at nearby locations and potentially suppressing additional vaporization and cavitation events. Such shielding effects from bubble clouds have been reported in other therapeutic-ultrasound contexts and provide a plausible basis for mixture conditions that yield lower emissions than a single-agent case at specific pressures [38].

Conversely, once the acoustic pressure is sufficient for acoustic droplet vaporization (ADV), mixtures can promote cavitation by increasing the effective number of bubbles generated. In this regime, pre-existing microbubbles can serve as readily activated nuclei, and the coexistence of gas bodies and phase-change droplets can facilitate the creation and maintenance of a more persistent cavitating population across pulses. Because ADV depends on threshold-like behavior and on nonlinear pressure-wave distortion/focusing, relatively small changes in the local acoustic environment can shift the balance between delayed activation and enhanced activation, providing a mechanistic rationale for observing both destructive and constructive mixture effects depending on the pressure range. Once droplets vaporize and additional bubbles are generated, bubble–bubble interactions can further modify collapse dynamics and emission spectra (e.g., clustering and collective behavior), which may amplify broadband emissions in some regimes while also increasing the likelihood of field attenuation in others. Thus, the mixture effect is expected to be inherently pressure-dependent, reflecting the transition from MB-dominated oscillations at lower pressures to ADV-enabled increases in bubble population at higher pressures, with shielding and collective effects becoming increasingly relevant as the bubble population grows [27], [39].

At lower PNP (1.2–3.1 MPa), cavitation activity is primarily governed by MB oscillations. This behavior is consistent with the low excitation threshold of gas-filled microbubbles, which readily undergo volumetric oscillations under relatively weak acoustic forcing [28], [40], [41]. In contrast, nanodroplets require sufficient negative pressure to trigger acoustic droplet vaporization, explaining their comparatively weaker cavitation response at low pressures. The reduced stable cavitation observed for the DFB MB + DFB ND mixture under some conditions suggests a possible destructive interaction between the two cavitation nuclei populations. As noted above, oscillating microbubbles can locally scatter the acoustic field, reducing the effective negative pressure experienced by nearby droplets and delaying their vaporization. Once the acoustic pressure increases sufficiently to activate droplet vaporization, the coexistence of MBs and newly formed bubbles from ND vaporization can increase the effective bubble population within the focal region, thereby promoting stronger inertial cavitation through bubble–bubble interactions and collapse dynamics [22]. In contrast, OFP ND cases exhibited relatively low stable cavitation levels, which may be attributed to the lower boiling point and reduced stability of OFP droplets, leading to earlier vaporization and rapid rupture compared with DFB droplets [15].

In the high PNP range (4.6–7.5 MPa), a distinctive cavitation trend was observed compared with that observed at lower pressures (1.2–3.1 MPa). At 4.6 MPa, both OFP ND and DFB ND cases exhibited higher SCL and FT amplitudes than those exhibited by DFB MB. This trend is consistent with previous studies showing that ND-mediated cavitation can produce stronger responses than MB-mediated cavitation under high-PNP pulsed ultrasound excitation [10], [26], [42]. The DFB MB + DFB ND mixture maintained high inertial cavitation activity, demonstrating that MB-ND synergy enhances bubble collapse and energy release. However, the lowest cavitation response was consistently observed in the DFB ND + OFP ND case, implying that droplet–droplet interactions do not significantly contribute to cavitation intensity despite the two distant vaporization thresholds may induce unique vaporization distributions at the focal volume.

At even higher pressures (6.3 MPa and 7.5 MPa), the DFB MB + DFB ND combination consistently exhibited the highest cavitation activity. The broader response and increased standard deviation in cavitation signals suggest that the cavitation process becomes more erratic at elevated pressures, with greater fluctuations in bubble dynamics. Notably, DFB MB alone recorded the lowest stable cavitation levels at these pressures, indicating that MBs may have reached a collapse threshold, leading to rapid depletion before sustaining long-term cavitation activity. This emphasizes the critical role of DFB NDs in enhancing cavitation at higher pressures, where MB instability becomes a limiting factor. Additionally, cases involving OFP ND including OFP ND alone, DFB MB + OFP ND, and DFB ND + OFP ND, exhibited the lowest ICL levels. This trend suggests that the inherently low stability and easy vaporization characteristics of OFP NDs offer no beneficial effects when high-PNP pulses are used. Instead, premature vaporization and rupture at physiological temperatures (∼36°C) and PNPs exceeding 6 MPa possibly lead to a reduced population of cavitation nuclei at the insonation focus, ultimately limiting cavitation activity.

Although inertial cavitation is expected to dominate at these high peak negative pressures, the concurrent enhancement of stable cavitation signatures observed in the DFB MB + DFB ND condition may still have mechanistic and practical significance. Stable oscillations of microbubbles can locally modify the acoustic pressure field and facilitate acoustic droplet vaporization of nearby nanodroplets, thereby increasing the effective bubble population within the focal region [16]. In this context, the observed stable cavitation activity should not be interpreted as an independent therapeutic objective under inertially dominated conditions. Rather, stable oscillations may serve as a seeding and sustaining mechanism that helps maintain a cavitation population capable of undergoing subsequent inertial collapse events. From a therapeutic standpoint, this interaction may contribute to a more persistent and spatially distributed cavitation cloud, enabling repeated inertial collapse events while mitigating rapid depletion of cavitation nuclei [43]. Therefore, the potential benefit of MB–ND combinations at high acoustic pressures lies not in independently maximizing both cavitation modes, but in promoting a cavitation environment in which stable oscillations facilitate the maintenance of bubble populations that ultimately drive inertial cavitation effects.

Previous studies have shown that increased acoustic pressure enhances stable and inertial cavitation activity of perfluorocarbon NDs [10], [26], [33]. In line with these findings, we initially anticipated a similar trend in this study. However, at very high pressures (7.5 MPa), inertial cavitation levels showed only slight variation compared to 6.3 MPa, suggesting a possible saturation effect [44]. This plateau is consistent with established cavitation dynamics, where excessive bubble activity at high pressures results in substantial ultrasound scattering and absorption, limiting further cavitation enhancement. Additionally, dense bubble clouds formed at high pressures can act as acoustic shields, reducing ultrasound penetration into the focal zone. Measurement variability also increases due to the erratic nature of cavitation at these levels, where intense forces cause bubble collapse or fragmentation, depleting bubbles available for sustained cavitation. These factors collectively contribute to a saturation point in cavitation response, consistent with previously described dynamics [45].

In relatively high PNP ranges (>3 MPa), DFB NDs exhibited greater inertial cavitation activity than OFP NDs under identical experimental conditions. This behavior can be attributed to several intrinsic properties of DFB. First, DFB has a higher boiling point (−1.7°C) compared with OFP (−36.7°C), requiring greater energy input for vaporization. Under high-pressure conditions, DFB droplets absorb more energy before transitioning into the gas phase, leading to the formation of larger, unstable bubbles prone to rapid collapse, hallmarks of inertial cavitation. Additionally, DFB remains in a superheated liquid state for a longer duration than OFP, allowing it to store more acoustic energy before vaporization. This superheated state predisposes DFB droplets to sudden, explosive vaporization, resulting in intense inertial cavitation as the bubbles collapse under the influence of a large force [16]. Furthermore, DFB’s higher latent heat of vaporization demands greater energy absorption during the gas phase transition, contributing to forceful bubble expansion and subsequent collapse, thereby intensifying inertial cavitation effects. These properties make DFB NDs more susceptible to inertial cavitation at high pressures, whereas OFP NDs vaporize more readily and tend to oscillate stably without violent collapse [46], [47].

Additionally, microbubbles and phase-change nanodroplets formulated with different perfluorocarbon cores can exhibit substantially different cavitation thresholds and persistence under ultrasound exposure because the physicochemical properties of the perfluorocarbon strongly influence their vaporization and stability. Previous studies have shown that microbubbles containing higher molecular weight perfluorocarbons such as perfluorobutane or perfluoropropane generally exhibit greater gas retention and circulation stability than air-filled bubbles because of their lower gas solubility and diffusivity in blood [13], [19]. Similarly, phase-change nanodroplets formulated with higher-boiling-point perfluorocarbons such as perfluoropentane (PFP) or perfluorohexane (PFH) typically require higher acoustic pressures to trigger vaporization but can remain metastable in the liquid state for longer durations under physiological conditions [20], [46]. In contrast, low-boiling-point droplets such as OFP or DFB vaporize more readily and therefore can be activated at lower acoustic pressures, although their post-vaporization bubble population may be less stable because of faster gas diffusion and bubble dissolution. Our observations are broadly consistent with previously reported trends in phase-change nanodroplet behavior. In several pressure conditions, DFB nanodroplets produced higher inertial cavitation levels than OFP nanodroplets, particularly at moderate to high excitation pressures. This tendency may reflect the greater metastability of DFB droplets and their ability to accumulate acoustic energy prior to vaporization. However, the distributions of cavitation levels overlapped across several treatments, and in some cases, mixtures involving OFP droplets exhibited comparable or higher responses. These results suggest that cavitation behavior depends not only on perfluorocarbon boiling point but also on interactions between droplets and pre-existing bubbles, local vaporization dynamics, and pressure-dependent bubble cloud formation.

Beyond the cavitation thresholds associated with different perfluorocarbon formulations, the stability of cavitation agents under physiological conditions is also an important consideration for potential biomedical applications. The composition of the lipid shell can also influence the performance and stability of microbubbles and nanodroplets. Variations in phospholipid structure, such as differences in hydrocarbon chain length, degree of saturation, and the presence of stabilizing components such as polyethylene glycol (PEG), can affect shell elasticity, permeability, and resistance to gas diffusion. These properties directly influence bubble stability, circulation persistence, and the ability of cavitation nuclei to withstand repeated acoustic oscillations. Consequently, both lipid shell composition and the choice of perfluorocarbon core contribute to the overall activation threshold and cavitation dynamics of MB–ND systems. In biological environments such as blood circulation, the persistence of cavitation nuclei is influenced by gas diffusion, shell permeability, temperature, and interactions with plasma proteins and cellular components. Microbubbles typically exhibit circulation lifetimes on the order of several minutes because gradual gas diffusion through the shell and mechanical fragmentation lead to progressive bubble dissolution [13]. In contrast, phase-change nanodroplets exist in a condensed liquid state prior to activation and therefore generally demonstrate improved stability and longer circulation times compared with gas-filled microbubbles [19]. When microbubbles and nanodroplets are combined, the mixture may benefit from complementary characteristics. Microbubbles can provide immediate cavitation nuclei that respond rapidly to ultrasound excitation, whereas nanodroplets may serve as a reservoir of cavitation nuclei that can be activated progressively through acoustic droplet vaporization. Such interactions may help maintain an active cavitation population even after initial microbubble collapse events. However, interactions between bubbles and droplets, including acoustic shielding, bubble coalescence, and rapid vaporization of low-boiling-point droplets, may also influence cavitation persistence depending on local acoustic pressure and droplet composition. Therefore, although the present in-vitro results demonstrate pressure-dependent cavitation behavior for MB–ND mixtures, further investigations under physiologically relevant conditions will be necessary to directly evaluate their stability and cavitation persistence in vivo.

Time-domain analyses revealed distinct cavitation signal patterns for each treatment. At lower pressures, cavitation signals exhibited an initial sharp peak followed by gradual decay, whereas at higher pressures, this peak became more pronounced, signifying intensified cavitation activity for the constant 10-cycle burst duration. DFB MB and OFP ND exhibited a distinct envelope of a secondary wave packet emerging at 65 μs, while DFB ND-based treatments displayed dominant initial peaks with low-amplitude, sustained cavitation signals (7% of the initial peak amplitude on average), including combination treatments. This suggests that DFB ND primarily induces cavitation through direct excitation bursts, whereas DFB MB and OFP ND facilitate secondary cavitation effects via scattered waves. Additionally, we confirmed that DFB MB maintained high cavitation amplitudes even at lower pressures, owing to its low rupture threshold (MI < 0.3), whereas both DFB and OFP NDs required higher pressures to achieve significant cavitation.

A key observation was the dominant inertial cavitation activity exhibited by the DFB MB + DFB ND combination at high PNP levels (6.3 MPa and 7.5 MPa), surpassing all other treatments. This case also produced the highest average time-domain signal amplitude, indicating a synergistic interaction between MBs and NDs. The enhanced cavitation response may be attributed to a larger cavitation cloud volume due to MB-assisted agitation, which facilitates the entire cavitation process of DFB NDs, encompassing vaporization, oscillation, rupture, and subsequent nucleation, within an expanded input ultrasound focus. Given that the excitation duration was only 10 μs (10 cycles of 1 MHz waves), the prolonged cavitation effects observed beyond 15 μs at the primary peak suggest extended cavitation activity beyond the initial excitation. When used together, NDs transform into MBs in situ, creating a dynamic system that possibly promotes cavitation across a broader pressure range. This integration can leverage the stability of NDs and the oscillation capability of MBs, leading to enhanced cavitation and improved control over therapeutic outcomes. Additionally, the MB-induced agitation environment may have facilitated the activation of more OFP ND particles via scattered waves, particularly in cases involving OFP ND alone, or in mixtures. The increasing STD of cavitation signals with rising pressure further reflects the growing variability and unpredictability of cavitation activity at higher acoustic intensities.

These findings provide insights into optimizing cavitation agents for therapeutic applications. The observed synergies in MB–ND mixtures suggest that combining these agents can maximize both stable and inertial cavitation effects at specific pressure ranges (>6 MPa). Interactions between microbubble oscillations and droplet vaporization may help maintain an active cavitation population within the focal region, thereby contributing to stronger inertial collapse events. Furthermore, the pressure-dependent variations in cavitation efficiency indicate the importance of tailoring ultrasound parameters to achieve desired therapeutic outcomes.

Although this study provides quantitative evidence of cavitation enhancement by MB–ND mixtures, certain limitations should be acknowledged. Specifically, cavitation behavior was characterized by using only one pulsing condition: 10-cycle, 1 MHz bursts delivered at a 2 Hz PRF (duty cycle 0.002%). As discussed, this low-duty cycle was chosen to ensure consistency of ultrasound excitations to flowing particles and cavitation signal acquisition while avoiding premature rupture under the experimental flow rate (80 ± 15 mL/min). Consequently, direct application of this pulsing scheme to therapeutic ultrasound modalities that typically employ longer duty cycles (>50%) [24], [25], [42], such as cavitation-enhanced HIFU for thermal ablation, biosample fragmentation, or drug delivery, would require further parameter optimization. Future work should therefore explore a broader range of pulse durations and duty cycles, using cavitation-level evaluations with similar PCD setups to refine pulsing strategies. Nonetheless, given that short-pulsed, high-pressure ultrasound has demonstrated significant advantages in recent histotripsy studies [48], [49], [50], our findings suggest that MB–ND mixtures could provide unique opportunities for developing more effective cavitation-enhanced strategies across diverse therapeutic ultrasound applications.

The present experiments were performed in a controlled, degassed aqueous flow model to enable mechanistic, pressure-resolved comparison of cavitation emissions between single agents and MB–ND mixtures. Commercially available clinical microbubble agents differ from in-house formulations in shell composition, core gas, size distribution, and concentration; these variables can substantially influence both harmonic and broadband cavitation emissions. Therefore, direct quantitative comparison of absolute SCL/ICL values across commercial products would require a dedicated experimental design with appropriate agent characterization and dose-matching strategies. In addition, protein-containing or blood-mimicking media and post-production storage time can affect microbubble stability and cavitation behavior. Future studies will extend the present analysis to serum-containing/blood-mimicking buffers and evaluate time-dependent changes in cavitation signatures to further assess translational performance [17], [51].

## 5 Conclusion

This study demonstrated the potential of combining DFB MBs and NDs to enhance cavitation activity across a broad range of acoustic pressures. Our hypothesis that the integration of MBs with low-boiling-point NDs would yield synergistic advantages beyond those of either agent alone was partially validated. The DFB MB + DFB ND mixture exhibited the most significant synergy at higher pressure regimes (>4.6 MPa), where MB instability becomes a limiting factor, and ND-mediated cavitation enhances overall efficacy. The interplay between DFB NDs and MBs optimizes cavitation efficiency by leveraging the stability of the NDs at high pressures and the oscillatory capacity of MBs at lower pressures. This dual-agent approach not only enhances stable cavitation at elevated acoustic pressures but also amplifies inertial cavitation across a broad pressure spectrum. Our findings provide experimental evidence of the complex interactions among MBs, NDs, and ultrasound excitation in regulating cavitation dynamics. Future research should focus on validating these interactions through microscopic single-particle analysis, investigating the long-term stability of these cavitation agents, and assessing their bioeffects in therapeutic applications. Further optimization of ultrasound parameters and agent formulations will be crucial for translating these findings into clinical applications.

## CRediT authorship contribution statement

**Haseeb Khan:** Writing – original draft, Formal analysis, Data curation. **Paul A. Dayton:** Writing – review & editing, Resources, Project administration, Funding acquisition. **Zhen Xu:** Writing – review & editing, Validation, Investigation. **Xiaoning Jiang:** Writing – review & editing, Methodology, Validation. **Jinwook Kim:** Writing – review & editing, Validation, Supervision, Project administration, Methodology, Investigation, Funding acquisition, Formal analysis, Data curation, Conceptualization, Resources.

## Declaration of competing interest

The authors declare the following financial interests/personal relationships which may be considered as potential competing interests: Paul A. Dayton is a co-founder of Triangle Biotechnology, a company that develops cavitation-enhancing agent technology. Jinwook Kim, Paul A. Dayton, Xiaoning Jiang, and Zhen Xu are co-inventors on patents involving nanodroplet applications including sonothrombolysis. Paul A. Dayton and Xiaoning Jiang have scientific advisory roles in SonoVascular, Inc. Zhen Xu has financial and/or other relationships with Histosonics, Inc., Ann Arbor, MI, USA.
