# Supplementary material for: Evaluation of cavitation enhancements in low-boiling point (< −2°C) perfluorocarbon nanodroplet and microbubble mixtures using therapeutic ultrasound pulses
Source: Ultrason Sonochem. 2026 Apr 13;129:107850. doi: 10.1016/j.ultsonch.2026.107850 (PMC13101723; doi:10.1016/j.ultsonch.2026.107850)
Supplement: Supplementary Data 1 [file mmc1.pdf]

Evaluation of Cavitation Enhancements in Low-boiling Point (<-2°C) Perfluorocarbon Nanodroplet and Microbubble Mixtures Using Therapeutic Ultrasound Pulses

Supplementary Material

S1. Spectral Analysis

To assess the overall spectral characteristics of the acoustic emissions and to verify the intrinsic frequency content of the recorded signals, the untreated fast Fourier transform (FFT) spectra were first examined. These spectra were computed directly from the raw time-domain signals without applying DC subtraction, bandpass filtering, smoothing, or normalization. Consequently, the untreated spectra represent the complete acoustic response generated during ultrasound exposure and includes the fundamental frequency, harmonic emissions, and broadband spectral components simultaneously. In Fig. S1, each panel corresponds to a single treatment, while the curves represent different peak negative pressure levels. This representation allows the pressure dependent spectral response of each formulation to be directly observed for the same treatment. However, because multiple spectral contributions overlap in the raw spectra, the cavitation regimes cannot be clearly distinguished at this stage. Therefore, additional preprocessing was performed to isolate the spectral regions associated with stable and inertial cavitation, which are presented in the main manuscript (Fig. 3 and Fig. 4).

S2. Statistical Analysis

To rigorously assess the differences in cavitation responses among various treatment conditions, we performed a comprehensive statistical analysis. In this study, three primary types of ultrasound-responsive agents microbubbles (MB), nanodroplets (ND), and their binary mixtures were investigated under identical experimental conditions. Specifically, we maintained a constant peak negative pressure (PNP) level, using the same setup, acquisition parameters, and data processing pipeline across all groups to ensure experimental uniformity. The only variation among the groups was the type or combination of cavitation nuclei introduced.

To assess whether the differences in cavitation responses among the treatment groups were statistically significant, we applied a one-way analysis of variance (ANOVA). This test is appropriate for comparing the means of three or more independent groups under the assumption of variance homogeneity. ANOVA was conducted at a significance level of $\boldsymbol{\alpha = 0.05}$, allowing us to test the null hypothesis that all groups share the same population mean.

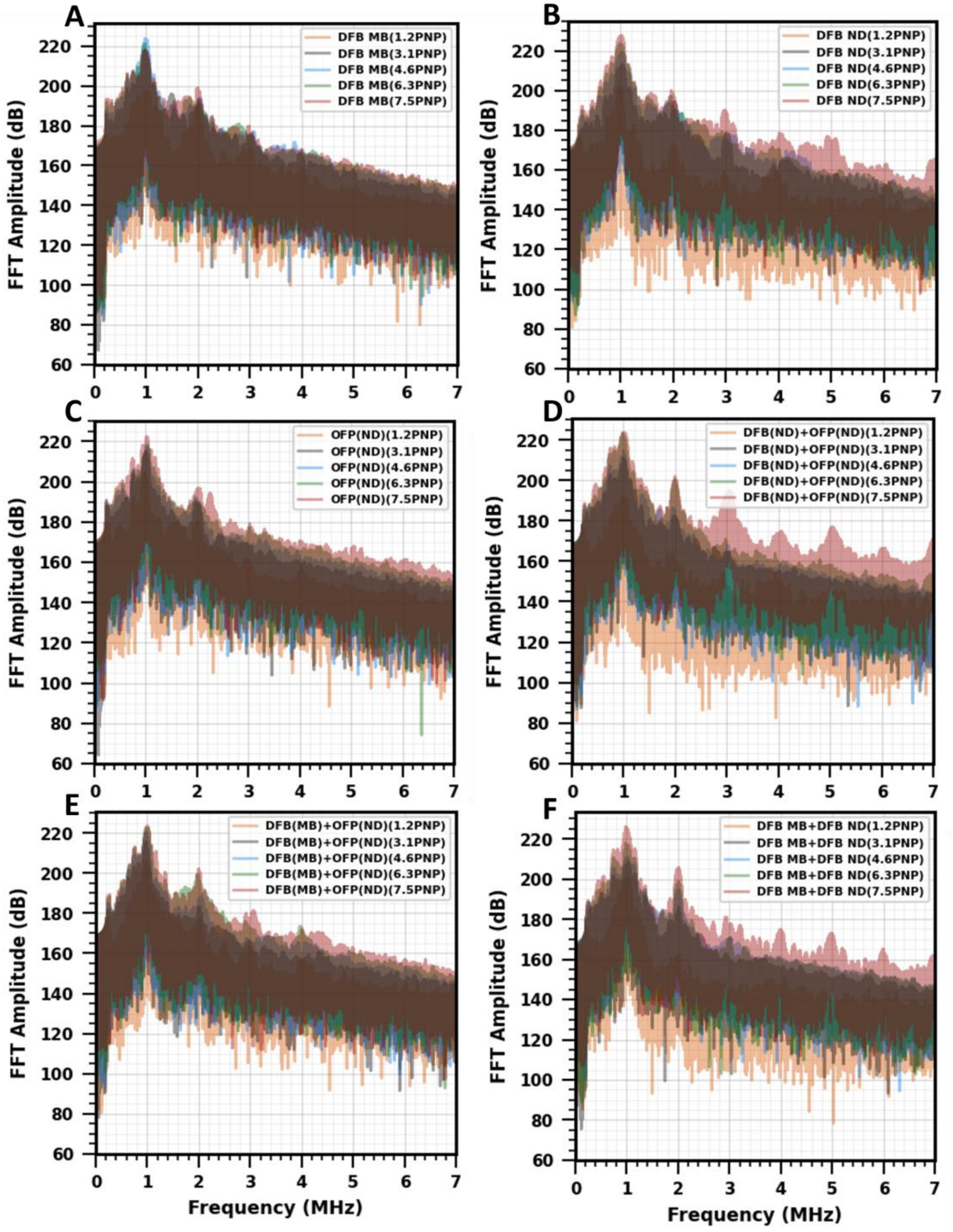

Fig. S1. Untreated FFT spectra of acoustic emissions for each treatment across different peak negative pressure levels.

Following a significant ANOVA result, we performed Tukey's Honest Significant Difference (HSD) test to identify which specific group pairs differed. Tukey's test was chosen because it effectively controls the family-wise error rate when making multiple comparisons between group means. In this study, we

analyzed six groups for each PNP value. To quantify the total number of unique pairwise comparisons among these six groups at each pressure level, we utilized Equation (S1).

$$c = \binom{n}{2} = \frac{n(n-1)}{2} \quad \text{(S1)}$$

For n = 6,

$$c = \binom{6}{2} = \frac{6(5)}{2} = 15 \quad \text{(S2)}$$

Thus, for each PNP level, a total of 15 unique pairwise comparisons were conducted to evaluate statistically significant differences in cavitation behavior across treatments.

All *p*-values obtained from these comparisons are comprehensively listed in Tables S1–S5 for stable cavitation and S6–S10 for inertial cavitation, corresponding to the five different PNP levels tested.

Table S1. Significance values of treatment effects corresponding to 1.2 MPa.

Label	DFB MB +OFP ND	DFB ND	DFB MB+ DFB ND	DFB ND + OFP ND	OFP ND
DFB MB	2.88E-04	2.60E-01	2.26E-04	4.52E-05	5.29E-08
DFB MB+OFP ND	N/A	1.07E-02	1.74E-07	6.51E-08	5.79E-10
DFB ND	N/A	N/A	1.18E-05	3.13E-06	9.65E-09
DFB MB+DFB ND	N/A	N/A	N/A	8.27E-01	4.02E-05
DFB ND +OFP ND	N/A	N/A	N/A	N/A	1.99E-04
OFP ND	N/A	N/A	N/A	N/A	N/A

Table S2. Significance values of treatment effects corresponding to 3.1 MPa.

Label	DFB MB + OFP ND	DFB ND	DFB MB + DFB ND	DFB ND + OFP ND	OFP ND
DFB MB	7.89E-03	5.19E-01	5.30E-02	2.55E-05	1.34E-07
DFB MB+OFP ND	N/A	1.48E-01	8.59E-01	1.78E-02	1.01E-05
DFB ND	N/A	N/A	6.37E-01	2.60E-04	6.31E-07
DFB MB+DFB ND	N/A	N/A	N/A	2.74E-03	2.94E-06
DFB ND +OFP ND	N/A	N/A	N/A	N/A	2.21E-03
OFP ND	N/A	N/A	N/A	N/A	N/A

Table S3. Significance values of treatment effects corresponding to 4.6 MPa.

Label	DFB MB+OFP ND	DFB ND	DFB MB+ DFB ND	DFB ND +OFP ND	OFP ND
DFB MB	1.47E-04	4.93E-05	1.53E-02	1.81E-07	6.99E-10
DFB MB+OFP ND	N/A	9.57E-01	8.95E-02	4.83E-04	9.81E-08
DFB ND	N/A	N/A	2.24E-02	1.70E-03	1.95E-07
DFB MB+DFB ND	N/A	N/A	N/A	9.76E-06	1.02E-08
DFB ND +OFP ND	N/A	N/A	N/A	N/A	5.53E-05
OFP ND	N/A	N/A	N/A	N/A	N/A

Table S4. Significance values of treatment effects corresponding to 6.3 MPa.

Label	DFB MB+OFP ND	DFB ND	DFB MB+DFB ND	DFB ND +OFP ND	OFP ND

DFB MB	8.90E-01	9.52E-04	4.50E-03	2.68E-04	5.86E-06
DFB MB+OFP ND	N/A	5.08E-03	2.63E-02	1.28E-03	1.97E-05
DFB ND	N/A	N/A	9.15E-01	9.49E-01	1.95E-02
DFB MB+DFB ND	N/A	N/A	N/A	4.77E-01	3.81E-03
DFB ND +OFP ND	N/A	N/A	N/A	N/A	8.27E-02
OFP ND	N/A	N/A	N/A	N/A	N/A

Table S5. Significance values of treatment effects corresponding to 7.5 MPa.

Label	DFB MB+OFP ND	DFB ND	DFB MB+DFB ND	DFB ND +OFP ND	OFP ND
DFB MB	2.74E-01	1.09E-02	8.40E-04	1.37E-04	6.53E-06
DFB MB+OFP ND	N/A	4.03E-01	3.42E-02	4.16E-03	1.05E-04
DFB ND	N/A	N/A	6.14E-04	1.14E-01	1.89E-03
DFB MB+DFB ND	N/A	N/A	N/A	8.00E-01	2.65E-02
DFB ND +OFP ND	N/A	N/A	N/A	N/A	2.06E-01
OFP ND	N/A	N/A	N/A	N/A	N/A

Table S6. Significance values of treatment effects corresponding to 1.2 MPa (Inertial Cavitations).

Label	DFB MB+OFP ND	DFB ND	DFB MB+DFB ND	DFB ND +OFP ND	OFP ND
DFB MB	2.73E-01	1.70E-04	2.27E-08	4.27E-06	7.78E-12
DFB MB+OFP ND	N/A	9.67E-06	4.59E-09	4.49E-07	1.67E-12
DFB ND	N/A	N/A	1.33E-05	8.75E-02	6.60E-10
DFB MB+DFB ND	N/A	N/A	N/A	7.36E-04	4.66E-07
DFB ND +OFP ND	N/A	N/A	N/A	N/A	4.71E-09
OFP ND	N/A	N/A	N/A	N/A	N/A

Table S7. Significance values of treatment effects corresponding to 3.1 MPa (Inertial Cavitation).

Label	DFB MB+OFP ND	DFB ND	DFB MB+DFB ND	DFB ND +OFP ND	OFP ND
DFB MB	4.90E-03	6.04E-02	3.79E-04	3.89E-07	9.01E-06
DFB MB+OFP ND	N/A	6.71E-01	5.83E-01	6.50E-05	6.58E-03
DFB ND	N/A	N/A	7.05E-04	1.04E-05	6.19E-04
DFB MB+DFB ND	N/A	N/A	N/A	6.53E-04	1.04E-01
DFB ND +OFP ND	N/A	N/A	N/A	N/A	7.49E-02
OFP ND	N/A	N/A	N/A	N/A	N/A

Table S8. Significance values of treatment effects corresponding to 4.6 MPa (Inertial Cavitation).

Label	DFB MB+OFP ND	DFB ND	DFB MB+DFB ND	DFB ND +OFP ND	OFP ND
DFB MB	5.01E-04	1.74E-06	2.35E-04	2.89E-07	9.88E-13
DFB MB+OFP ND	N/A	5.78E-03	9.94E-01	3.03E-04	6.01E-11
DFB ND	N/A	N/A	1.39E-02	4.42E-01	1.13E-09
DFB MB+DFB ND	N/A	N/A	N/A	6.55E-04	8.39E-11
DFB ND +OFP ND	N/A	N/A	N/A	N/A	3.94E-09
OFP ND	N/A	N/A	N/A	N/A	N/A

Table S9. Significance values of treatment effects corresponding to 6.3 MPa (Inertial Cavitation).

Label	DFB MB+OFP ND	DFB ND	DFB MB+DFB ND	DFB ND +OFP ND	OFP ND
DFB MB	2.08E-02	1.33E-05	6.62E-06	3.44E-04	3.55E-08
DFB MB+OFP ND	N/A	2.79E-03	1.06E-03	1.74E-01	9.58E-07
DFB ND	N/A	N/A	9.93E-04	1.97E-01	3.97E-04
DFB MB+DFB ND	N/A	N/A	N/A	7.45E-02	9.88E-01
DFB ND +OFP ND	N/A	N/A	N/A	N/A	1.50E-05
OFP ND	N/A	N/A	N/A	N/A	N/A

Table S10. Significance values of treatment effects corresponding to 7.5 MPa (Inertial Cavitation).

Label	DFB MB+OFP ND	DFB ND	DFB MB+DFB ND	DFB ND +OFP ND	OFP ND
DFB MB	7.42E-01	7.02E-01	2.29E-04	2.12E-01	1.65E-02
DFB MB+OFP ND	N/A	1.00E+00	8.99E-01	8.79E-01	1.60E-01
DFB ND	N/A	N/A	9.23E-05	9.05E-01	1.78E-01
DFB MB+DFB ND	N/A	N/A	N/A	1.00E+00	6.06E-01
DFB ND +OFP ND	N/A	N/A	N/A	N/A	6.37E-01
OFP ND	N/A	N/A	N/A	N/A	N/A

To interpret the findings summarized in Tables S1–S10, we examined the patterns of statistical significance across pressure levels for both stable and inertial cavitation. The resulting trends offer key insights into how treatment combinations respond differently under varying acoustic intensities.

At 1.2 MPa, both stable and inertial cavitation levels varied distinctly across treatment groups, with several pairwise comparisons showing statistically significant differences (Fig. 2A in the main paper, Supplementary Tables S1 and S6). Specifically, DFB MB produced significantly higher cavitation than DFB ND, OFP ND, and the mixture DFB ND + OFP ND ($p < 0.001$), highlighting the superior cavitation efficiency of microbubbles at lower acoustic pressures. In contrast, although DFB MB + OFP ND showed moderately elevated cavitation levels, it did not differ significantly from OFP ND or DFB ND + OFP ND ($p > 0.05$) in either mode. This suggests a potential suppressive or neutralizing effect of OFP nanodroplets when co-administered with microbubbles under low energy conditions, possibly due to changes in bubble dynamics or shielding effects.

Similarly, at 3.1 MPa, both stable and inertial cavitation levels became more pronounced across all formulations shown in Fig. 2 (A and B), and the number of statistically significant comparisons increased accordingly (Tables S2 and S7). In the stable cavitation regime, DFB MB maintained a consistently higher cavitation level than DFB ND, OFP ND, and DFB ND + OFP ND ($p < 0.05$), reaffirming the dominant role of microbubbles under moderate pressure. However, formulations like DFB MB + OFP ND and DFB MB + DFB ND remained statistically comparable to each other ($p > 0.05$), indicating that the inclusion of nanodroplets did not significantly enhance or impair the stable cavitation response at this stage. In the inertial cavitation mode, DFB MB and its binary mixtures (with OFP ND or DFB ND) exhibited markedly higher cavitation levels compared to nanodroplet-only groups ($p < 0.01$), confirming their greater

responsiveness under increasing pressure. Still, certain mixed-agent formulations such as DFB MB + OFP ND vs. DFB MB + DFB ND remained statistically indistinguishable, suggesting that while microbubble presence drives inertial cavitation, the type of nanodroplet combined with it has limited influence on energy release at this intermediate pressure.

At 4.6 MPa, cavitation activity became substantially more dynamic referring to Fig. 2, as both amplitude levels and statistical separation across groups increased. In the stable cavitation regime (Table S3), although DFB MB remained significantly different from all other groups ($p < 0.01$), it was not the highest-performing formulation. Interestingly, OFP ND alone showed the highest stable cavitation level, exceeding even MB-based mixtures. This suggests that, under mid-range pressures, OFP nanodroplets undergo sufficient vaporization and bubble oscillation to rival or surpass MB-induced stability. Despite this performance, DFB MB + OFP ND and DFB MB + DFB ND still differ significantly from OFP ND and DFB ND + OFP ND, confirming that the presence of MBs drives a statistically distinct response pattern even when the overall cavitation level is not the absolute highest. Similarly, in the inertial cavitation regime (Table S8), the combination of DFB MB + DFB ND emerged as the most active formulation, exhibiting the highest inertial energy output. Most pairwise comparisons were statistically significant ($p < 0.01$), reflecting clear separation among treatment groups. However, the comparison between DFB MB + OFP ND and DFB MB + DFB ND remained non-significant (p = 0.99), suggesting these two mixed-agent strategies exhibit comparable inertial cavitation behaviors despite their compositional differences. These findings collectively indicate that, at 4.6 MPa, both ND-only and MB–ND combinations start to show competitive and distinguishable cavitation behavior, influenced by the balance of vaporization potential and bubble dynamics in each formulation.

At elevated acoustic pressures of 6.3 MPa and 7.5 MPa, the mixture of DFB MB + DFB ND consistently produced the highest cavitation levels in both stable and inertial cavitation, as illustrated in Figs. 2A and 2B, respectively. This trend was not only visually evident but also strongly supported by the statistical significance analyses presented in Supplementary Tables S4–S5 (stable) and Tables S9–S10 (inertial).

At 6.3 MPa, in the stable cavitation mode (Table S4), DFB MB + DFB ND demonstrated statistically significant differences from multiple nanodroplet-based treatments, including DFB ND + OFP ND ($p = 0.0038$) and OFP ND ($p = 0.0038$). This indicates that the high cavitation levels seen in Fig. 2A are not due to random variation but reflect a consistent improvement over ND-based groups. In the inertial cavitation case (Table S9), DFB MB + DFB ND again showed statistically significant separation from DFB MB ($p = 6.62E\text{-}06$) and DFB ND ($p = 9.93E\text{-}04$), confirming that the addition of nanodroplets further enhances cavitation beyond MB-only performance.

At the highest tested pressure of 7.5 MPa, this synergistic trend became even more distinct. In stable cavitation (Table S5), DFB MB + DFB ND differed significantly from DFB ND + OFP ND ($p = 0.0265$) and DFB MB ($p = 0.00084$), consistent with its peak response observed in Fig. 2A. In inertial cavitation (Table S10), DFB MB + DFB ND again demonstrated significant differences compared to DFB MB ($p = 2.29E\text{-}04$), DFB ND ($p = 9.23E\text{-}05$), and DFB ND + OFP ND ($p = 0.606$), aligning with the peak inertial activity in Fig. 2B.

These statistically validated pairwise comparisons reinforce that DFB MB + DFB ND consistently induces significantly stronger cavitation at high pressures than any other formulation tested, both visually and quantitatively. The convergence of graphical trends (Fig. 2) and statistical outcomes (Tables S4–S10) emphasizes the robust enhancement of cavitation achieved by combining microbubbles and nanodroplets, supporting its potential for therapeutic ultrasound applications where maximum acoustic activation is desired.

S3. Analysis of cavitation signals in the time domain

We also identified the time-domain patterns of the cavitation signal. To achieve this, we conducted two types of analyses: first, we examined the same treatment at different PNP pressures (1.2, 3.1, 4.6, 6.3, and 7.5 MPa) and we observed that as the PNP pressure increased, the primary peak of the cavitation signal consistently became more pronounced, and its amplitude increased considerably. This peak was observed consistently in the vicinity of the 40 μs mark across all conditions, highlighting the direct correlation between PNP pressure and cavitation intensity. At the highest pressure of 7.5 MPa, the peak reached its maximum value, showcasing the substantial enhancement in energy release due to higher pressure. Additionally, we noticed that secondary peaks (which occurred between 60 and 80 μs) were more distinctive in some test groups than others. These peaks displayed varied amplitudes depending on the PNP applied, indicating a potential influence of residual bubble or droplet dynamics post cavitation. Treatments such as DFB MB, DFB ND, and OFP ND exhibited distinct secondary peak patterns, emphasizing the role of individual treatment components in shaping the signal as outlined in the main text (Fig. 5). However, following integration of microbubbles (MBs) and nanodroplets (NDs) under the same PNP conditions, we observed a shift in the signal dynamics, where the primary peaks became broader, and the secondary peaks displayed altered intensities and timings (Fig. 5). This suggests that the combined interactions of MBs and NDs induce synergistic effects, enhancing and modifying overall cavitation behavior. The integrated treatments, such as DFB MB + OFP ND, demonstrated a prolonged signal decay compared with the response induced by single-agent treatments, indicating more sustained cavitation activity. In the second analysis, we examined the same pressure under different treatments, as shown in Fig. S2. We noticed that at 1.2 MPa, the MB DFB exhibits comparable amplitudes in the DFB MB+OFP ND and DFB ND+OFP

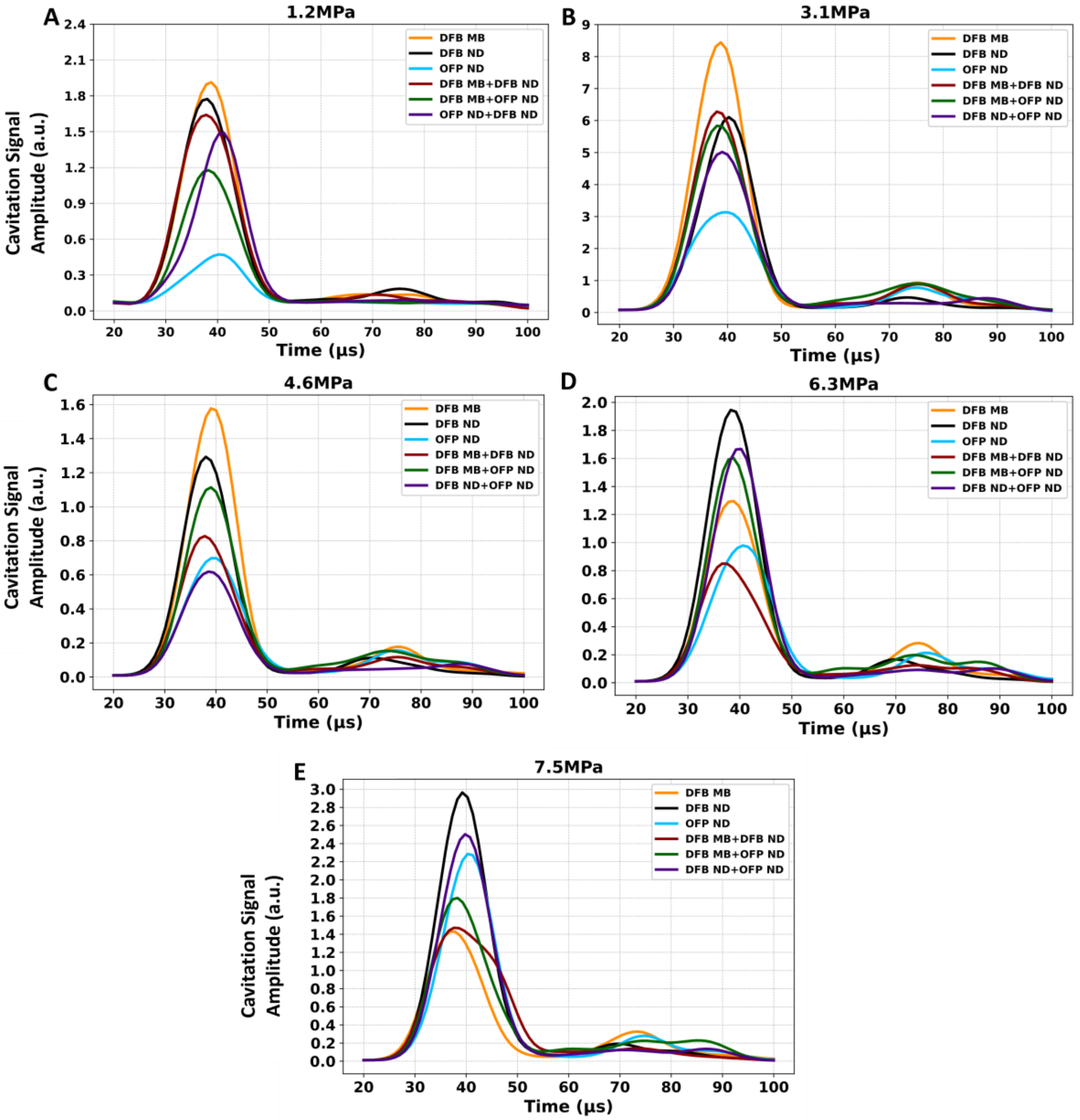

Fig. S2. Time-domain signal comparisons for the various treatments applied herein at the same pressure level.

ND cases. However, at 3.1 MPa and 4.6 MPa, the MBs yield higher amplitudes than those induced when MBs and NDs are integrated, as shown in Fig. S2 [B and C].

These findings provide a deeper understanding of the pressure-dependent dynamics of cavitation signals, demonstrating the unique response characteristics of DFB MB and highlighting the increasing variability of cavitation signals at higher pressures. The inclusion of these observations in the supplementary materials provides a detailed context useful for interpreting the trends and mechanisms underlying the results presented in the main text.
